# 13.1 ENDOCANNABINOID MODULATION OF DOPAMINE NEUROTRANSMISSION

**DOI:** 10.1093/schbul/sby014.049

**Published:** 2018-04-01

**Authors:** Fabricio Moreira

**Affiliations:** Federal University of Minas Gerais

## Abstract

**Background:**

Dopamine is the major neurotransmitter implicated in schizophrenia pathology. Thus, understanding the processes modulating dopaminergic signalling may lead to new insights in the biology and treatment of this disorder. The endocannabinoids anandamide and 2-arachidonoylglicerol (2-AG) modulate neural activity through interactions CB1 and CB2 receptors. CB1 antagonists inhibit the effects of drugs that facilitate dopamine activity, such as cocaine. Similar to CB1 antagonists, CB2 agonists counteract the effects of cocaine in experimental animals. However, the functions of these receptors have been investigated separately. Here we test the hypothesis that CB1 and CB2 receptors interact to ameliorate the behavioural and molecular processes altered under hyperdopaminergic states. We also sought to identify the endocannabinoid involved in these effects.

**Methods:**

Male Swiss mice received cocaine injections to increase dopamine activity in the brain. The biological responses measured were hyperlocomotion, conditioned place preference, cFos expression and Erk protein phosphorylation in the nucleus accumbens. The animals received cannabinoid-related drugs before cocaine injections. The data were analysed with ANOVA followed by the Newman-Keuls test.

**Results:**

The CB1 receptor antagonist, rimonabant, and the CB2 receptor agonist, JWH133, inhibited cocaine-induced hyperlocomotion. Moreover, the CB2 antagonist, AM630, reversed the inhibitory effects of rimonabant in cocaine-induced hyperlocomotion, cFos expression, Erk phosphorylation and conditioned place preference. The inhibitors of anandamide and 2-AG hydrolysis, URB597 (FAAH inhibitor) and JZL184 (MGL inhibitor), respectively, were ineffective in inhibiting cocaine hyperlocomotion. However, when combined with a sub-effective dose of rimonabant, JZL184 (but not URB597), inhibited cocaine effects.

**Discussion:**

A CB2 antagonist reversed the effect of a CB1 antagonist, suggesting that these receptors modulate cocaine effects in opposite ways. Accordingly, increasing brain 2-AG levels inhibited cocaine effects only if CB1 is blocked and CB2 available. Thus, selective activation of CB2 receptors warrants further investigation as a new strategy for the treatment of psychiatric disorders resulting from hyperdopaminergic states.

